# SNNRice6mA: A Deep Learning Method for Predicting DNA N6-Methyladenine Sites in Rice Genome

**DOI:** 10.3389/fgene.2019.01071

**Published:** 2019-10-11

**Authors:** Haitao Yu, Zhiming Dai

**Affiliations:** ^1^School of Data and Computer Science, Sun Yat-Sen University, Guangzhou, China; ^2^Guangdong Province Key Laboratory of Big Data Analysis and Processing, Sun Yat-Sen University, Guangzhou, China

**Keywords:** epigenetics, deep learning, DNA sequence, rice, bioinformatics

## Abstract

DNA N6-methyladenine (6mA) is an important epigenetic modification, which is involved in many biology regulation processes. An accurate and reliable method for 6mA identification can help us gain a better insight into the regulatory mechanism of the modification. Although many experimental techniques have been proposed to identify 6mA sites genome-wide, these techniques are time consuming and laborious. Recently, several machine learning methods have been developed to identify 6mA sites genome-wide. However, there is room for the improvement on their performance for predicting 6mA sites in rice genome. In this paper, we developed a simple and lightweight deep learning model to identify DNA 6mA sites in rice genome. Our model needs no prior knowledge of 6mA or manually crafted sequence feature. We built our model based on two rice 6mA benchmark datasets. Our method got an average prediction accuracy of ∼93% and ∼92% on the two datasets we used. We compared our method with existing 6mA prediction tools. The comparison results show that our model outperforms the state-of-the-art methods.

## Introduction

DNA N6-methyladenine (6mA) is one important DNA epigenetic modification, which has been found in bacteria, eukaryote, and archaea (O’brown and Greer, 2016). It was reported that 6mA is involved in many biological processes. For example, in bacteria, 6mA plays roles in DNA replication (Campbell and Kleckner, 1990), DNA repair (Au et al., 1992), transcription (Robbins-Manke et al., 2005), and gene expression regulation (Low et al., 2001). But for eukaryote, the study of DNA 6mA”” is still insufficient (Koziol et al., 2016). Studying the distribution of DNA 6mA can provide a deeper understanding of the epigenetic modification process. 

Recently, the development of experimental techniques contributes to studying DNA 6mA modification. Pormraning et al. developed a protocol using bisulfite sequencing and methyl-DNA immunoprecipitation technique to analyze genome-wide DNA methylation in eukaryotes (Pomraning et al., 2009). Krais et al. reported a fast and sensitive method for the quantification of global adenine methylation in DNA, using laser-induced fluorescence and capillary electrophoresis (Krais et al., 2010). Flusberg et al. applied single-molecule, real-time sequencing technique to detect DNA methyladenine directly (Flusberg et al., 2010). Greer et al. used ultra-high performance liquid chromatography coupled with mass spectrometry technique to access DNA 6mA levels in *Caenorhabditis elegans* (Greer et al., 2015).

Due to the large cost of experiment-based approach, researchers have used machine learning technology to identify and predict DNA 6mA sites. Feng et al. proposed a SVM-based tool (called iDNA6mA-PseKNC) to predict 6mA sites in *Mus musculus* genome (Feng et al., 2019). Feng et al. validated iDNA6mA-PseKNC on the *M. musculus* dataset and other 6mA datasets of eight microbe species. It was reported that iDNA6mA-PseKNC achieved very high prediction accuracy on all the nine datasets, revealing that this method is reliable and can identify genome-wide 6mA sites in many species. Recently, Chen et al. provided a benchmark 6mA dataset containing 880 6mA sites and 880 non-6mA sites in rice genome (Chen et al., 2019), which was denoted as 6mA-rice-Chen in this study. By using many manually crafted DNA sequence features, they built a support vector machine (SVM) based tool (called i6mA-Pred) to identify 6mA sites in rice genome. It was reported that i6mA-Pred got an accuracy of ∼83% on the rice genome dataset. Pian et al. proposed a tool, called MM-6mAPred, based on the markov model for 6mA sites prediction (Pian et al., 2019). Pian et al. built and evaluated their MM-6mAPred based on the 6mA-rice-Chen benchmark dataset. It was reported that MM-6mAPred outperformed i6mA-Pred in prediction of 6mA sites. Tahir et al. proposed another computational tool, called iDNA6mA, for 6mA identification in rice genome (Tahir et al., 2019). Tahir et al. trained and evaluated their iDNA6mA on the 6mA-rice-Chen dataset, and they found that iDNA6mA outperformed i6mA-Pred in prediction performance. Basith et al. proposed a tool, named SDM6A, for predicting 6mA sites in the rice genome (Basith et al., 2019). SDM6A is an ensemble approach using several features encoding methods and machine learning classifiers. Basith et al. trained and evaluated their SDM6A based on the 6mA-rice-Chen benchmark dataset, and they found that SDM6A outperformed i6mA-Pred and iDNA6mA on the 6mA-rice-Chen benchmark dataset. Very recently, Lv et al. proposed a computational tool, iDNA6mA-rice, for prediction of 6mA sites in rice genome (Lv et al., 2019). Lv et al. proposed another 6mA benchmark dataset for rice genome, and we denoted such dataset as 6mA-rice-Lv. The 6mA-rice-Lv contains 154,000 6mA sites-contained sequences as the positive samples and the same number of negative samples. Lv et al. trained and evaluated iDNA6mA-rice on 6mA-rice-Lv dataset by five-fold cross-validation, and they found that iDNA6mA-rice achieved good prediction performance. For the purpose of the comparison with i6mA-Pred, Lv et al. also trained and evaluated iDNA6mA-rice on the 6mA-rice-Chen dataset and found that iDNA6mA-rice outperformed i6mA-Pred on the 6mA-rice-Chen dataset.

Previous studies have shown that deep learning is a powerful technique for sequences analysis and classification in bioinformatics (Zhang et al., 2018; Zou et al., 2019). In this paper, we proposed a simple, lightweight, and high-performance method to improve prediction accuracy of DNA 6mA sites in rice genome (called SNNRice6mA). SNNRice6mA is based on convolutional neural network architecture. It needs no manually crafted sequence feature and can learn high level abstract features, compared with traditional machine learning based methods. SNNRice6mA got an accuracy of ∼93% and ∼92% on the 6mA-rice-Chen and 6mA-rice-Lv datasets, respectively. SNNRice6mA performed better than previous methods in prediction of DNA 6mA sites in rice genome.

## Methods

### Dataset

In this study, we considered two 6mA benchmark datasets for rice genome. The first dataset is the 6mA-rice-Chen dataset (Chen et al., 2019), which was widely used by previous studies (Basith et al., 2019; Chen et al., 2019; Pian et al., 2019; Tahir et al., 2019). The 6mA-rice-Chen dataset contains 880 positive samples and 880 negative samples. The second dataset we used is the 6mA-rice-Lv dataset (Lv et al., 2019). The 6mA-rice-Lv dataset contains 154,000 positive samples and 154,000 negative samples. DNA sequences in both positive samples and negative samples are 41 bp long. For each positive sequence, its center is the 6mA modification site. For each negative sequence, its center contains no 6mA modification site. By using these two widely used datasets, we can compare our method with previous methods fairly.

### The SNNRice6mA Method

#### Data Representation

The samples in our dataset are DNA sequences, expressed in a string form. For example, a sample is like “GTATAT… GCCTAA.” Before feeding the sequences to the model, we should first convert the sequence into encoding tensor. 

Previous methods, including i6mA-Pred, iDNA6mA-PseKNC, SDM6A, and iDNA6mA-rice, used manually crafted sequences features to represent DNA sample sequences, such as nucleotide chemical properties and nucleotide frequency (Basith et al., 2019; Feng et al., 2019; Lv et al., 2019). Manually crafted sequences features require a large amount of prior knowledge of DNA 6mA modification and may be unsuitable for large data size. 

Instead of using manually crafted DNA sequences features, we used the one-hot encoding method. A, T, C, and G are encoded as (1,0,0,0), (0,1,0,0), (0,0,1,0), and (0,0,0,1), respectively. Each sample sequence in our dataset is 41 bp long. After one-hot encoding, each sequence is converted to a matrix, which has 41 columns and 4 rows. Each column of the matrix represents a specific DNA base of the sample sequence. In brief, the information fed to our model is only the base composition of a sample sequence, without any manually crafted DNA sequences feature. 

#### Model Details

We built a deep learning method, called SNNRice6mA, based on the rice genome benchmark datasets. The architecture of our method is a typical convolutional neural network. SNNRice6mA contains eight components, constructed in a stacked way. The input vector of SNNRice6mA is a one-hot encoding DNA sequence. The first component of SNNRice6mA is a one-dimensional convolution layer, which is abbreviated as Conv. The layer Conv has 16 filters, whose lengths are all 4. Every filter in the layer Conv functions like a sequence motif recognizer of 6mA modification sites in rice genome. For each input vector, each filter searches sequence patterns that can discriminate true 6mA containing sequences from pseudo ones. To avoid overfitting, we used the L2 regularization method for the filter weights and bias in Conv layer. And we set all regularization rates as 0.0001. The activation function used in Conv layer is the exponential linear unit (ELU) activation function. The second component of SNNRice6mA is a group normalization layer (GN), which aims to reduce the correlation of the results produced by each filter in Conv layer. Group normalization is suitable for the small size of the training batch (Wu and He, 2018). The GN divides the outputs of Conv layer into some groups and carries out the normalization in each group, respectively. We set the number of groups as four in GN. The third component of SNNRice6mA is a one-dimensional max pooling layer, reducing the redundancy of the features that the previous layer outputs. We set the size of the max pooling windows as 4, which is the same as the size of the filter in convolution layer. We used the dropout layer after the pooling layer. The dropout layer acts like a regularization function to prevent overfitting of the model. In each training iteration, the dropout layer randomly shuts down some hidden neurons units by setting the outputs of these units to zero. So, after the dropout process, some intermediate features are discarded, which prevents overfitting and can improve the reliability and robustness of the model. We set the dropout rate as 0.25. After dropout layer, we used a flatten function to integrate the intermediate features, which are fed to the fully connected (FC) layer. The FC layer has 32 hidden units. To avoid overfitting, we used the L2 regularization method for the weights and bias. And we set all regularization rates as 0.0001. The activation function used in FC layer is ELU activation function. The output of FC layer is fed to the last component, sigmoid function. The sigmoid function outputs a float value between 0 and 1, which is considered as the probability of the input DNA sequence containing 6mA modification site. If the probability is larger than 0.5, the model will classify the input DNA sequence as the positive sample, which means the center of input DNA sequence is the 6mA site. If the probability is smaller than 0.5, the discrimination is the opposite.

We used the optimizer, stochastic gradient descent (SGD) with momentum, and the binary cross-entropy loss function. We set the learning rate as 0.005 and the momentum rate as 0.95 in SGD optimizer. We set the maximum training epoch as 100 and the batch size of training as 32. We used early stopping technique in the training process. The early stopping means that the training process will stop when the prediction accuracy stops improving on the validation set. We set patience of early stopping as 30 epochs, which means that the training is stopped when the prediction accuracy on validation set does not improve after 30 training epochs. We also used the model checkpoint technique, which saves the model which has the highest prediction accuracy on the validation set. During the training process, we reduced the learning rate when the value of loss function on validation set no longer decreased. We set the reduced factor as 0.1 and the patience as 20 epochs, which means that the learning rate is reduced when the value of loss function on validation set does not improve after 20 training epochs.

We implemented our method based on Keras 2.2.4. We used the default values of hyper-parameters in Keras, except those that have been mentioned in this paper (see the full list in **Table S1**).

### Performance Metrics

To be consistent with previous studies (Lv et al., 2019; Pian et al., 2019), we used the standard 10-fold cross-validation method to evaluate our method on the 6mA-rice-Chen dataset and used the standard 5-fold cross-validation method to evaluate our method on the 6mA-rice-Lv dataset. For example, in 10-fold-cross-validation, we randomly partitioned the rice genome benchmark dataset into 10 folds with equal size. In each cross-validation iteration, we used eight folds for training, one fold for validating, and the remaining one fold for testing. In each iteration, we saved the specific model with highest accuracy on the validation fold and evaluated the performance of this model on testing fold. The cross-validation iteration was executed 10 times, and the average predicted accuracy of 10 iterations was calculated. Our source codes are available on https://github.com/yuht4/SNNRice6mA. 

For the evaluation metrics, we used the same metrics as those in a previous study (Chen et al., 2019). Totally, five metrics have been used, including accuracy, sensitivity, specificity, Matthews correlation coefficient (MCC), and area under the curve (AUC).

The metric accuracy means the ratio of correct predictions on the testing data. The accuracy is defined as below:

$$\text{accuracy}=\frac{\mathrm{TP}+\mathrm{TN}}{\mathrm{TP}+\mathrm{TN}+\mathrm{FP}+\mathrm{FN}}$$

True positive (TP) is the number of predictions that classify the TP samples correctly. True negative (TN) is the number of predictions that classify the TN samples correctly. False positive (FP) is the number of predictions that misclassify the negative samples as the positive ones. False negative (FN) is the number of predictions that misclassify the positive samples as the negative ones. The positive means the samples containing the 6mA sites, and vice versa.

The metric sensitivity is the ratio of correctly identified positive samples in all actual positive data. The sensitivity is defined as below:

$$\mathrm{sensitivity}=\frac{\mathrm{TP}}{\mathrm{TP}+\mathrm{FN}}$$

The metric specificity is the ratio of correctly identified negative samples in all actual negative data. The specificity is defined as below:

$$\mathrm{specificity}=\frac{\mathrm{TN}}{\mathrm{TN}+\mathrm{FP}}$$

MCC is a measure of the quality of binary classification model (Matthews, 1975). MCC takes TP, TN, FP, and FN into account. MCC is generally regarded as a balanced measure that can be used even if the samples are unbalanced in two classes (Boughorbel et al., 2017).

The MCC measures the correlation between the real and predicted binary classifications. MCC is a coefficient value between −1 and +1. A value of +1 represents a perfect binary classification model, 0 means the same as random prediction, and −1 indicates total disagreement between predicted labels and real labels.

MCC is defined as below:

$$\mathrm{MCC}=\frac{\mathrm{TP}\times \mathrm{TN}-\mathrm{FP}\times \mathrm{FN}}{\sqrt{\left(\mathrm{TP}+\mathrm{FP})(\mathrm{TP}+\mathrm{FN})(\mathrm{TN}+\mathrm{FP})(\mathrm{TN}+\mathrm{FN}\right)}}$$

AUC is the area value under the receiver operating characteristic curve. AUC is also an important indicator to measure the performance of a binary classification model. The larger the AUC value, the better the performance of model. AUC is a float value between 0 and 1; 1 means the model is perfect in prediction, while 0.5 means the model is similar with random predictions.

## Results

**Figure 1:**
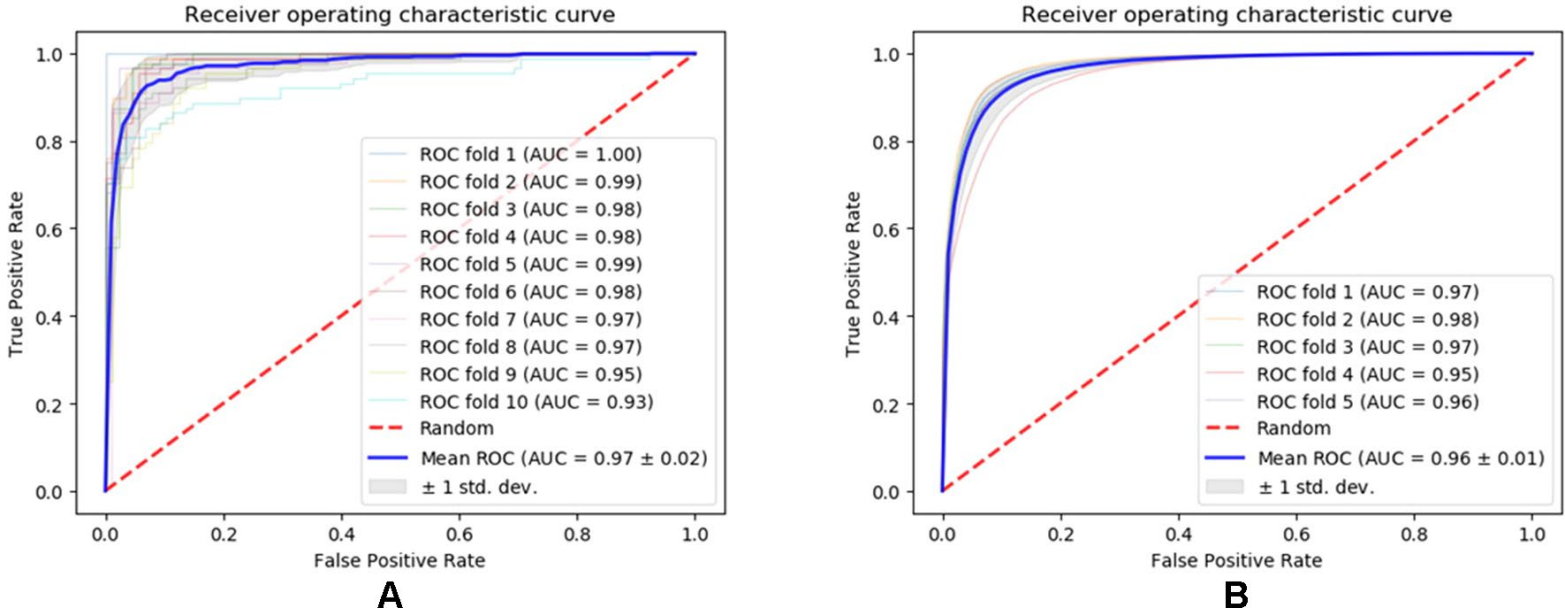
Receiver operating characteristic curves of SNNRice6mA on testing sets of 6mA-rice-Chen dataset and 6mA-rice-Lv dataset. **(A)** Performance on the 6mA-rice-Chen dataset. **(B)** Performance on the 6mA-rice-Lv dataset.

We evaluated the performance of our method SNNRice6mA on two DNA 6mA benchmark datasets (i.e., 6mA-rice-Chen and 6mA-rice-Lv) for rice genome. SNNRice6mA showed good performance on these two datasets in terms of different evaluation metrics (**Figures 1**, **S1, S2 and Tables S2, S3**). We compared SNNRice6mA with state-of-the-art tools. Results showed that SNNRice6mA performed better than these tools.

### Performance Comparison on Rice 6mA Benchmark Datasets

To the best of our knowledge, there are six existing tools for identifying DNA 6mA sites, including i6mA-Pred (Chen et al., 2019), iDNA6mA-PseKNC (Feng et al., 2019), SDM6A (Basith et al., 2019), iDNA6mA (Tahir et al., 2019), MM-6mAPred (Pian et al., 2019), and iDNA6mA-rice (Lv et al., 2019). Among them, i6mA-Pred, SDM6A, iDNA6mA, MM-6mAPred, and iDNA6mA-rice were all built based on the 6mA sites data in rice genome, which could predict the 6mA sites in rice genome. In addition, iDNA6mA-PseKNC is a tool built based on *M. musculus* dataset and can be applied in many other species (Feng et al., 2019). We examined whether iDNA6mA-PseKNC can predict 6mA sites in rice genome. We used the rice benchmark 6mA-rice-Chen dataset to test the performance of iDNA6mA-PseKNC and found that the error rate of iDNA6mA-PseKNC prediction is relatively high (∼58%). In this study, we thus just compared our method SNNRice6mA with the remaining five existing tools, including i6mA-Pred, SDM6A, iDNA6mA, MM-6mAPred, and iDNA6mA-rice. To be consistent with the evaluation metrics used in these studies, we used five metrics, including accuracy, sensitivity, specificity, MCC, and AUC.

Firstly, we compared SNNRice6mA with previous tools on the 6mA-rice-Chen dataset. The performance results of i6mA-Pred, SDM6A, iDNA6mA, MM-6mAPred, and iDNA6mA-rice were directly quoted from previous studies (Basith et al., 2019; Chen et al., 2019; Lv et al., 2019; Pian et al., 2019; Tahir et al., 2019). Note that the AUC value of MM-6mAPred was not calculated in the original study (Pian et al., 2019). We found that SNNRice6mA outperformed 6mA-Pred, SDM6A, iDNA6mA, MM-6mAPred, and iDNA6mA-rice in terms of sensitivity, specificity, accuracy, MCC, and AUC (**Table 1**).

**Table 1:** Performance comparison between SNNRice6mA and several previous methods on 6mA-Rice-Chen dataset.

Method	Sensitivity (%)	Specificity (%)	Accuracy (%)	MCC	AUC
SNNRice6mA	92.16	94.32	93.24	0.87	0.97
SNNRice6mA -feature	90.34	92.95	91.65	0.83	0.98
i6mA-Pred	82.95	83.30	83.13	0.66	0.89
MM-6mAPred	89.32	90.11	89.72	0.79	/
iDNA6mA	86.70	86.59	86.64	0.73	0.93
SDM6A	85.20	90.90	88.10	0.76	0.94
iDNA6mA-rice	83.86	83.41	83.63	0.67	0.91

6mA, N6-methyladenine; AUC, area under the curve; MCC, Matthews correlation coefficient.

Secondly, we compared SNNRice6mA with iDNA6mA-rice on the 6mA-rice-Lv dataset. During the peer reviews of our manuscript, Lv et al. proposed iDNA6mA-rice and released the 6mA-rice-Lv dataset (Lv et al., 2019). We used 5-fold cross-validation in training SNNRice6mA on the 6mA-rice-Lv dataset, which is the same validation strategy as that of iDNA6mA-rice (Lv et al., 2019). The performance of iDNA6mA-rice on the 6mA-rice-Lv dataset was directly quoted from the original study (Lv et al., 2019). We found that SNNRice6mA outperformed iDNA6mA-rice in only one of the five evaluation metrics (i.e., prediction sensitivity) (**Table 2**). Considering that the 6mA-rice-Lv dataset contains much more sample sequences than 6mA-rice-Chen dataset (308,000 vs. 1,760), we sought to examine whether increasing the model complexity can improve the prediction performance of SNNRice6mA on 6mA-rice-Lv dataset. We changed the number of filters to 32 and the number of hidden units in FC layer to 64. We denoted this complex version of SNNRice6mA as SNNRice6mA-large. We retrained SNNRice6mA-large on the 6mA-rice-Lv dataset. We found that SNNRice6mA-large outperformed original SNNRice6mA in all the five evaluation metrics, and SNNRice6mA-large outperformed iDNA6mA-rice in three of the five evaluation metrics (**Table 2**).

**Table 2:** Performance comparison between SNNRice6mA and iDNA6mA-Rice on 6mA-Rice-Lv dataset.

Method	Sensitivity (%)	Specificity (%)	Accuracy (%)	MCC	AUC
SNNRice6mA	93.67	86.74	90.20	0.81	0.96
SNNRice6mA-large	94.33	89.75	92.04	0.84	0.97
iDNA6mA-rice	93.00	90.50	91.70	0.84	0.96

6mA, N6-methyladenine; AUC, area under the curve; MCC, Matthews correlation coefficient.

### Comparison With Feature-Based Sequence Encoding Strategy

To examine the effect of sequence encoding scheme, we built another model, SNNRice6mA-feature, the same as SNNRice6mA except that SNNRice6mA-feature is built based on feature-based sequence encoding. The feature-based sequence encoding we used is the same as that in a previous study (Chen et al., 2019). Two sequence features have been considered, nucleotide chemical property and nucleotide frequency. The four DNA bases, adenine (A), thymine (T), cytosine (C), and guanine (G), have different chemical properties. (1) C and G can from hydrogen bonds strongly, while A and T form hydrogen bonds weakly. (2) A and G are purines, while T and C are pyrimidines. (3) A and C are amino groups, while T and G are keto groups. We can distinguish four DNA bases in three ways, including hydrogen bond strength, base type, and amino/keto group category. 

We used a triad to encode the chemical properties of four DNA bases. The first element of the triad indicates the base type; 1 means purines, and 0 means pyrimidines. The second element of the triad indicates the hydrogen bond strength; 1 means weak, while 0 means strong. The third element of the triad indicates the amino/keto group category; 1 means amino, and 0 means keto. So, we encoded A, T, C, and G as (1, 1, 1), (0, 1, 0), (0, 0, 1) and (1, 0, 0), respectively.

We used the same way as Chen et al. to calculate nucleotide frequency of every position in a sequence. The calculation formula is defined as below:

$$d_{1}=\frac{1}{L_{1}}\sum_{j=1}^{i}f(N_{j}),\;f(N_{j})=\{\begin{array}{l} 1,{\text{ if N}}_{j}\text{ is the nucleotide concerned}\\ 0,\text{ otherwise} \end{array}$$

where *d* *_i_* is the nucleotide frequency of position *i* in a DNA sequence. *L* *_i_* is the length of the subsequence from the first position to the position *i* of the sequence. *N* *_i_* stands for the base in position *i* of a DNA sequence (i.e., one of the A, T, C, and G).

Combining the nucleotide chemical properties and nucleotide frequency together, each DNA sequence can be represented as a matrix, with 41 columns and 4 rows. Each column of the matrix represents a specific DNA base. For each column, the first three elements represent the nucleotide chemical properties, and the last element represents its nucleotide frequency.

We trained SNNRice6mA-feature on the 6mA-rice-Chen dataset by using the feature-based sequence encoding method above. SNNRice6mA-feature was trained and tested in the same way as SNNRice6mA. SNNRice6mA-feature outperformed SNNRice6mA in only one of the five evaluation metrics (**Table 1**).

### Cross-Species Evaluation

We next tested whether model trained on rice datasets can be used to predict DNA 6mA sites in other species. We used the *M. musculus* 6mA dataset proposed in a previous study (Feng et al., 2019) and denoted this dataset as 6mA-mouse-Feng. 6mA-mouse-Feng dataset contains 1,934 6mA site containing sequences and 1,934 non-6mA site containing sequences. We used this independent dataset as test data. We evaluated the performance of SNNRice6mA, which was trained on rice 6mA-rice-Lv dataset, on the *M. musculus* test data. We also performed similar evaluation for three of the five rice 6mA prediction methods, including i6mA-Pred (Chen et al., 2019), iDNA6mA (Tahir et al., 2019), and MM-6mAPred (Pian et al., 2019). For the remaining two rice 6mA prediction methods, SDM6A (Basith et al., 2019) and iDNA6mA-rice (Lv et al., 2019), we encountered errors when using these two tools (**Figures S3, S4**). We thus excluded these two methods for evaluation. We found that SNNRice6mA achieved predicted accuracy of 61.81%, which was higher than those of the other three methods (52.43% for i6mA-Pred, 41.93% for iDNA6mA, 44.11% for MM-6mAPred).

## Conclusions

In this study, we proposed a deep learning model SNNRice6mA to identify DNA 6mA sites in rice genome. SNNRice6mA got similar predicted accuracies on the two rice datasets (∼93% and ∼92%). SNNRice6mA performed better than previous methods in prediction of 6mA sites. Though the limited size of available training dataset might bias the generalization of the model, we used some techniques (e.g., regularization) to minimize this artifact. We expect that our method can facilitate the analysis of DNA 6mA sites in the rice genome. However, there are some limitations for our method. First, the feature ranking is not possible in the current version. Second, there is room for improvement on the performance of rice data-trained SNNRice6mA on *M. musculus* dataset.

## Data Availability Statement

Publicly available datasets were analyzed in this study. These data can be found here: https://github.com/yuht4/SNNRice6mA. 

## Author Contributions

HY and ZD designed the study, analyzed the results, and drafted the manuscript. HY implemented the algorithms and carried out the experiments.

## Funding

This work was supported by National Natural Science Foundation of China (NSFC) (grant 61872395, U1611265), by Natural Science Foundation of Guangdong Province (2018A030313285), and also by Pearl River Nova Program of Guangzhou (201710010044).

## Conflict of Interest

The authors declare that the research was conducted in the absence of any commercial or financial relationships that could be construed as a potential conflict of interest.
