# Supplementary material for: SNNRice6mA: A Deep Learning Method for Predicting DNA N6-Methyladenine Sites in Rice Genome
Source: Front Genet. 2019 Oct 11;10:1071. doi: 10.3389/fgene.2019.01071 (PMC6797597; doi:10.3389/fgene.2019.01071)
Supplement: Supplementary file 1 [file DataSheet_1.pdf]

# *Supplementary Materials*

## 1 Supplementary Figures

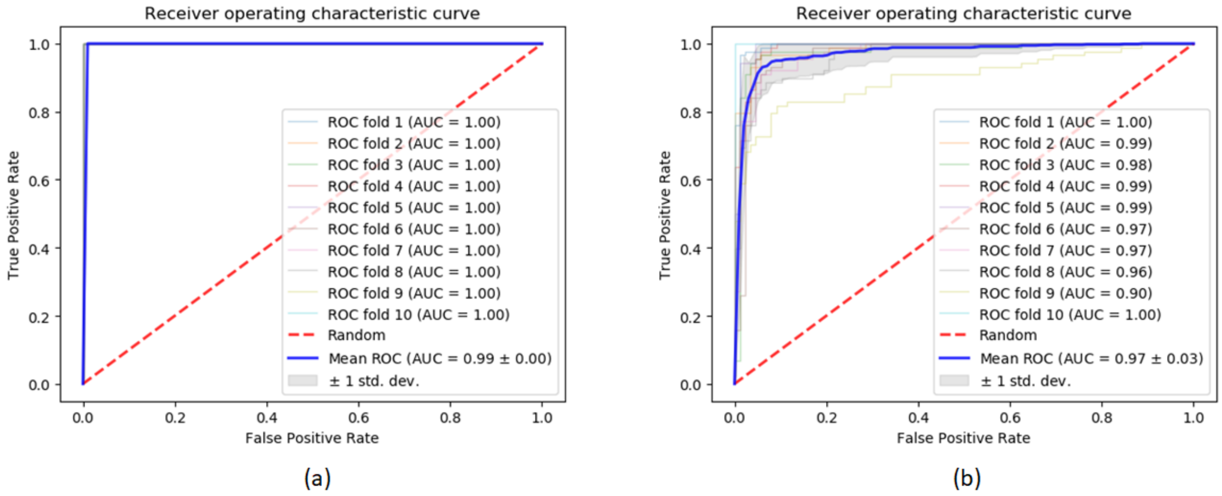


**Figure** S**1.** ROC curves of SNNRice6mA on training set and validation set of 6mA-rice-Chen dataset. (a) The performance on the training set. (b) The performance on the validation set.

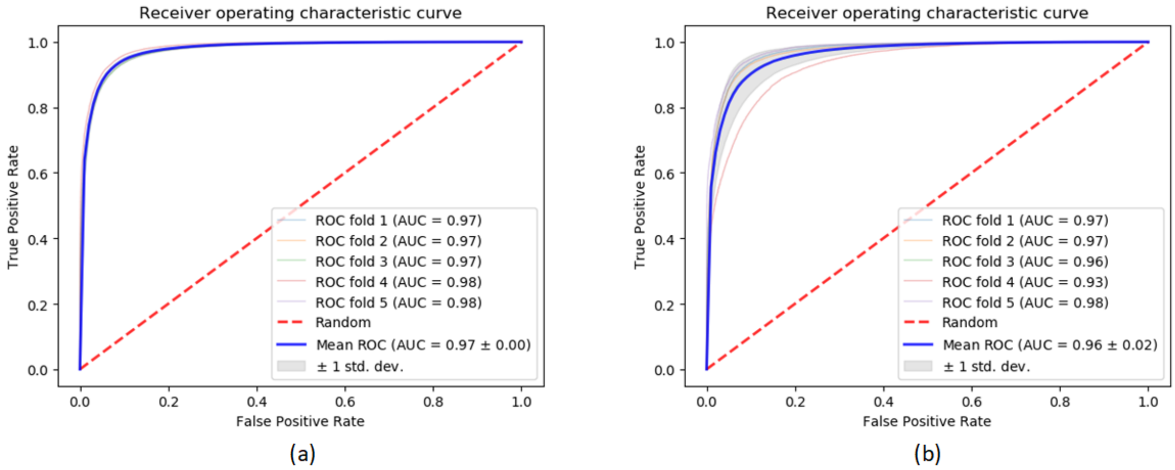


**Figure** S2. ROC curves of SNNRice6mA on training set and validation set of 6mA-rice-Lv dataset. (a) The performance on the training set. (b) The performance on the validation set.

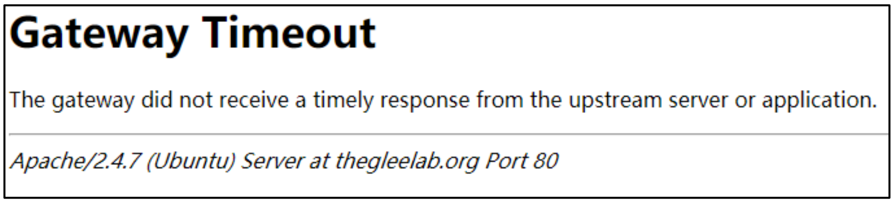


Figure S3 Error message when using the SDM6A tool on 6mA-mouse-Feng dataset (http://thegleelab.org/SDM6A/index.html).

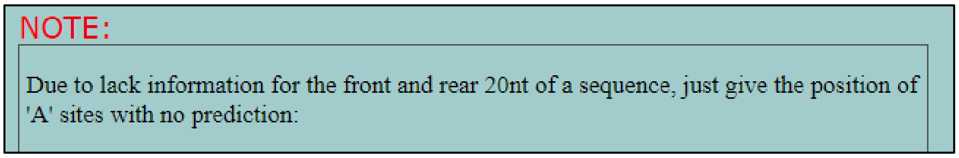


Figure S4 Error message when using the iDNA6mA-Rice tool on 6mA-mouse-Feng dataset (http://lin-group.cn/server/iDNA6mA-Rice/service.html).

## 2 Supplementary Tables

Table S1 Hyper-parameters of SNNRice6mA

| Hyper-parameter | Value |
|---|---|
| Number of filters | 16 |
| Kernel size of filter | 4 |
| Number of groups in GN layer | 4 |
| Regularization rate | 0.0001 |
| Dropout rate | 0.25 |
| Hidden units in FC layer | 32 |
| Learning rate | 0.005 |
| Momentum rate | 0.95 |
| Batch size | 32 |

Table S2 Performance of SNNRice6mA on 6mA-rice-Chen dataset

| Metric | Training set | Validation set | Testing set |
|---|---|---|---|
| Sensitivity (%) | 99.94±0.09 | 92.84±6.14 | 92.16±5.76 |
| Specificity (%) | 99.99±0.04 | 93.75±3.22 | 94.32±2.87 |
| Accuracy (%) | 99.96±0.07 | 93.30±4.10 | 93.24±3.71 |
| MCC | 1.00±0.00 | 0.87±0.08 | 0.87±0.07 |
| AUC | 0.99±0.00 | 0.97±0.03 | 0.97±0.02 |
| Precision (%) | 99.99±0.04 | 93.68±3.21 | 94.19±2.92 |
| F1-Score (%) | 99.97±0.07 | 93.19±4.31 | 93.09±3.91 |
| Binary cross entropy Loss | 0.01±0.01 | 0.21±0.13 | 0.22±0.12 |

Table S3 Performance of SNNRice6mA on 6mA-rice-Lv dataset

| Metric | Training set | Validation set | Testing set |
|---|---|---|---|
| Sensitivity (%) | 94.01±0.41 | 93.68±0.36 | 93.67±0.41 |
| Specificity (%) | 90.66±0.52 | 86.18±6.05 | 86.74±3.93 |
| Accuracy (%) | 92.34±0.44 | 89.93±3.10 | 90.20±1.92 |
| MCC | 0.85±0.01 | 0.80±0.06 | 0.81±0.04 |
| AUC | 0.97±0.00 | 0.96±0.02 | 0.96±0.01 |
| Precision (%) | 90.96±0.49 | 87.40±4.61 | 87.71±3.07 |
| F1-Score (%) | 92.46±0.42 | 90.37±2.62 | 90.56±1.64 |
| Binary cross entropy Loss | 0.20±0.01 | 0.25±0.07 | 0.25±0.04 |
